# Botulinum hemagglutinin-mediated selective removal of cells deviating from the undifferentiated state in hiPSC colonies

**DOI:** 10.1038/s41598-017-00083-1

**Published:** 2017-03-07

**Authors:** Mee-Hae Kim, Yo Sugawara, Yukako Fujinaga, Masahiro Kino-oka

**Affiliations:** 1 Department of Biotechnology, Graduate School of Engineering, Osaka University, 2-1 Yamadaoka, Suita, Osaka 565-0871 Japan; 2 Laboratory for Infection Cell Biology, International Research Center for Infectious Diseases, Research Institute for Microbial Diseases, Osaka University, 2-1 Yamadaoka, Suita, Osaka 565-0871 Japan; 3 Department of Bacteriology, Graduate School of Medical Sciences, Kanazawa University, 13-1 Takara, Kanazawa, Ishikawa 920-8640 Japan

## Abstract

The undifferentiated state of human induced pluripotent stem cells (hiPSCs) depends on their cell–cell and cell–substrate adhesions. In this study, we report that exposure to botulinum hemagglutinin (HA), an E-cadherin function-blocking agent, selectively removed cells that deviated from the undifferentiated state in hiPSC colonies. After HA treatment, cell–cell adhesion was disrupted, deviated cells detached from colony centers, and dividing cells filled these spaces. Because E-cadherin-mediated adhesion was disrupted in undifferentiated cells, stress-fiber formation and focal adhesions were diminished; however, these were subsequently restored, and the cells retained expression of undifferentiated stem cell markers and their differentiation potential. In contrast, actin structures and focal adhesions were lost from deviated cells, and they subsequently died. In undifferentiated and deviated cells, the cadherin/integrin-regulator Rap1 was localized at cell–cell adhesions and in the cytoplasm, respectively. Concurrent HA and Rap1-inhibitor treatment accelerated the deviated-cell detachment and delayed the recovery of hiPSC morphology, but this effect was significantly attenuated by co-treatment with Rap1 activator. Thus, Rap1 regulated E-cadherin–integrin interplay in hiPSC colonies exhibiting deviation, while HA-mediated selective removal of these deviated cells helped maintain the undifferentiated state in the remaining hiPSCs.

## Introduction

Human pluripotent stem cells (hPSCs), including human embryonic stem cells (hESCs) and human induced pluripotent stem cells (hiPSCs) hold great promise for clinical and industrial applications because they can self-renew and differentiate into all cell types^1, 2^. Although the methods to optimize hPSC expansion and differentiation have advanced considerably, numerous technological challenges remain^3–7^. Transitionally, hESC and hiPSC culture methods require the use of mouse or human fibroblast feeder layers, or feeder-conditioned medium. From these cultures, hPSCs spontaneously deviate from the undifferentiated state, a widely recognized phenomenon, whereby their morphology changes drastically into large flattened cells^8^. Upon prolonged culturing, cells in the deviated region invade and occupy the colony, thereby accelerating loss of their self-renewal ability and pluripotency during subculturing. hPSC cultures invariably exhibit a small amount of differentiation, and the cultures must be routinely cleaned by manually removing differentiated cells to prevent the differentiated areas from initiating morphological changes that can trigger colony-wide differentiation. Because these methods rely on the capabilities of researchers, maintaining the undifferentiated state in subcultures requires consistent and robust methods for eliminating deviated cells from hPSC cultures.

The cell–cell adhesion is primarily mediated by the E-cadherin and its function has been shown to be important in many aspects of cell state or differentiation^9–11^. This dynamic structure physically connects neighboring cells, couples intercellular adhesive contacts to the cytoskeleton, and helps define each cell’s apical–basal axis of polarity^12–15^. Whereas E-cadherin disruption can alter actin organization and focal adhesion, aberrant actin organization can cause changes in the cell-adhesion status. Several Ras- or Rho-family GTPases function at key intersections of the signaling pathways that control the interplay between cell–cell and cell–substrate adhesion^14–20^. The Ras-family GTPase Rap1 has emerged as an intriguing candidate coordinator of the spatiotemporal regulation of integrin- and cadherin-mediated adhesion^16–20^. This coordinated integrin-cadherin interplay, coupled with a loss of cadherin function, regulates the physical interaction between integrin- and cadherin-mediated adhesions. In hPSC cultures, E-cadherin-mediated cell–cell adhesion induces changes in both cell and colony morphologies, which potentially activates signaling pathways involved in either maintaining the undifferentiated state or committing to a lineage^21–30^. Recent studies reported that like keratinocytes, hESCs colonies exhibit structural characteristics of polarized epithelial cells including E-cadherin-mediated cell–cell adhesions and integrin-mediated cell–substrate adhesions^24–26^. They clearly demonstrated that the E-cadherin structure physically connects neighboring cells, couples intercellular adhesion to the cytoskeleton, and helps define each cells apical–basal axis. In addition, in hESC cultures, Rap1 affects the endocytic recycling pathway involved in the formation and maintenance of E-cadherin-mediated cell–cell adhesion, which is essential for the colony formation and self-renewal^29^. These findings provide insight into successful strategies for the regulating the hPSC self-renewal and differentiation^23^.

Regulation of E-cadherin-mediated cell–cell adhesion might have implications for improving drug delivery through the paracellular pathway of biological barriers (intestinal mucosa and the blood–brain barrier), and for understanding the mechanisms of cadherin-mediated interactions at intercellular junctions^31–34^. Clostridium botulinum hemagglutinin (HA) is a component of the large botulinum neurotoxin complex, and is critical for its oral toxicity. HA plays multiple roles in toxin penetration in the gastrointestinal tract, including protection from the digestive environment, binding to the intestinal mucosal surface, and disruption of the epithelial barrier^31^. It has become clear that the HA possess a potent ability to disrupts epithelial barrier function and have distinct features in their modes of action. HA is functionally and structurally separable into 2 parts: HA1, which is involved in recognizing cell-surface carbohydrates; and HA2–HA3, which is involved in paracellular-barrier disruption through E-cadherin binding^31^. HA directly binds E-cadherin in adherens junctions and disrupts E-cadherin-mediated cell–cell adhesion. HA treatment has been proposed as a method of disrupting epithelial barrier function^31–34^. These studies not only will provide an important insight into the mechanisms of these epithelial barrier disrupting activities but also may lead to unique and powerful opportunities to understand the complicated mechanisms for the stem cell function and maintenance.

Here, we investigated the mechanism by which HA exposure affects the removal of cells that deviate from the undifferentiated state in hiPSC colonies and the accompanying disruption of E-cadherin binding. Based on the observed differences in morphological behaviors between undifferentiated and deviated regions in single hiPSC colonies, we discuss the fundamental mechanisms of cell–cell and cell–substrate adhesion in relation to hiPSC survival, self-renewal, and death.

## Results

### Differences in E-cadherin expression level and cell morphology between the undifferentiated and deviated regions inside hiPSC colonies exhibiting deviation

**Figure 1:**
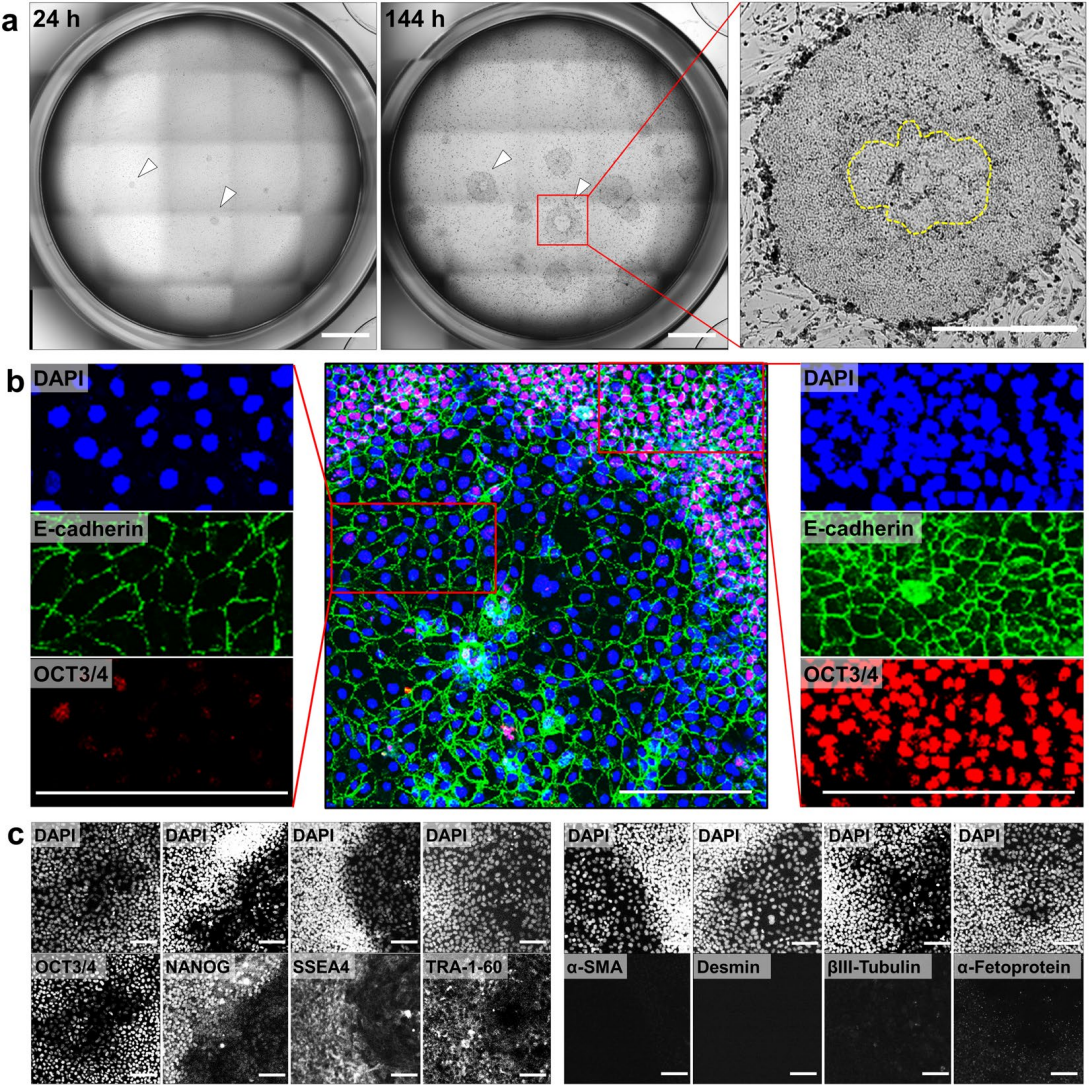
Characterization of hiPSC colonies exhibiting deviation in culture with SNL feeder cells. (**a**) Morphological properties of hiPSC colonies exhibiting deviation in 144-h cultures prepared with SNL feeder cells. The images show the occurrence of spontaneous deviation from the undifferentiated state of hiPSCs in an entire culture well of a 24-well plate. Solid arrowheads indicate the hiPSC colonies exhibiting deviation in the central regions. Dotted lines indicate the boundary between undifferentiated region and deviated region in hiPSC colonies. Scale bars = 1 mm. (**b**) Immunofluorescent staining for OCT3/4 (red) and E-cadherin (green). Cell nuclei were stained with DAPI (blue). Scale bars = 200 μm. (**c**) Immunofluorescent staining for four undifferentiated stem-cell markers (OCT3/4, NANOG, SSEA-4, TRA-1-60) and three germ-layer markers (α-Smooth muscle actin (α-SMA), Desmin, βIII-Tubulin, α-Fetoprotein). Cell nuclei were stained with DAPI. Scale bars = 100 μm.

hiPSCs were cultured with SNL feeder cells for 144 h, and following which hiPSC pluripotency and early lineage commitment were characterized by immunostaining. Undifferentiated hiPSC colonies subsequently expanded gradually and became more tightly packed, while all colonies containing only undifferentiated hiPSCs displayed a small cobblestone-like shape (Fig. 1a). In some colonies cultured with SNL feeder cells, hiPSCs deviated from the undifferentiated state and transformed into large flattened cells at the colony center (Fig. 1a, arrowheads). Prolongation of the culture was associated with expansion of both regions inside a single colony. To verify the undifferentiated region and deviated region in hiPSC colonies, staining of E-cadherin and OCT3/4 was applied to distinguish the deviation from the undifferentiated state in hiPSCs. In OCT3/4-expression cells at the colony periphery, E-cadherin was observed as a continuous line at the boundaries between neighboring cells. When the peripheral region inside a single colony was compared, however, the deviated cells at the central region of the colony exhibited disorganised or reduced E-cadherin expression, showing negative OCT3/4 (Fig. 1b). We further compared the markers expression of the undifferentiated stem cells (OCT3/4, NANOG, SSEA-4, TRA-1-60) and the three germ layers (mesoderm, endoderm, ectoderm). The deviated cells at the central region of the colony was negative in all undifferentiated stem cell markers as well as three germ layer markers (Fig. 1c). Thus, in this study, transformation of hiPSCs from the undifferentiated state to the deviated state in colonies was designated “deviation”, indicating that they lose their undifferentiated state, although it is hard to conclude the fully differentiated state. In other studies using hESC lines, however, the larger flattened cells at the central region of the colony sometimes appear to represent the committed extraembryonic endoderm lineage^22, 35^.

### HA effects on deviated-cell removal from hiPSC colonies exhibiting deviation

**Figure 2:**
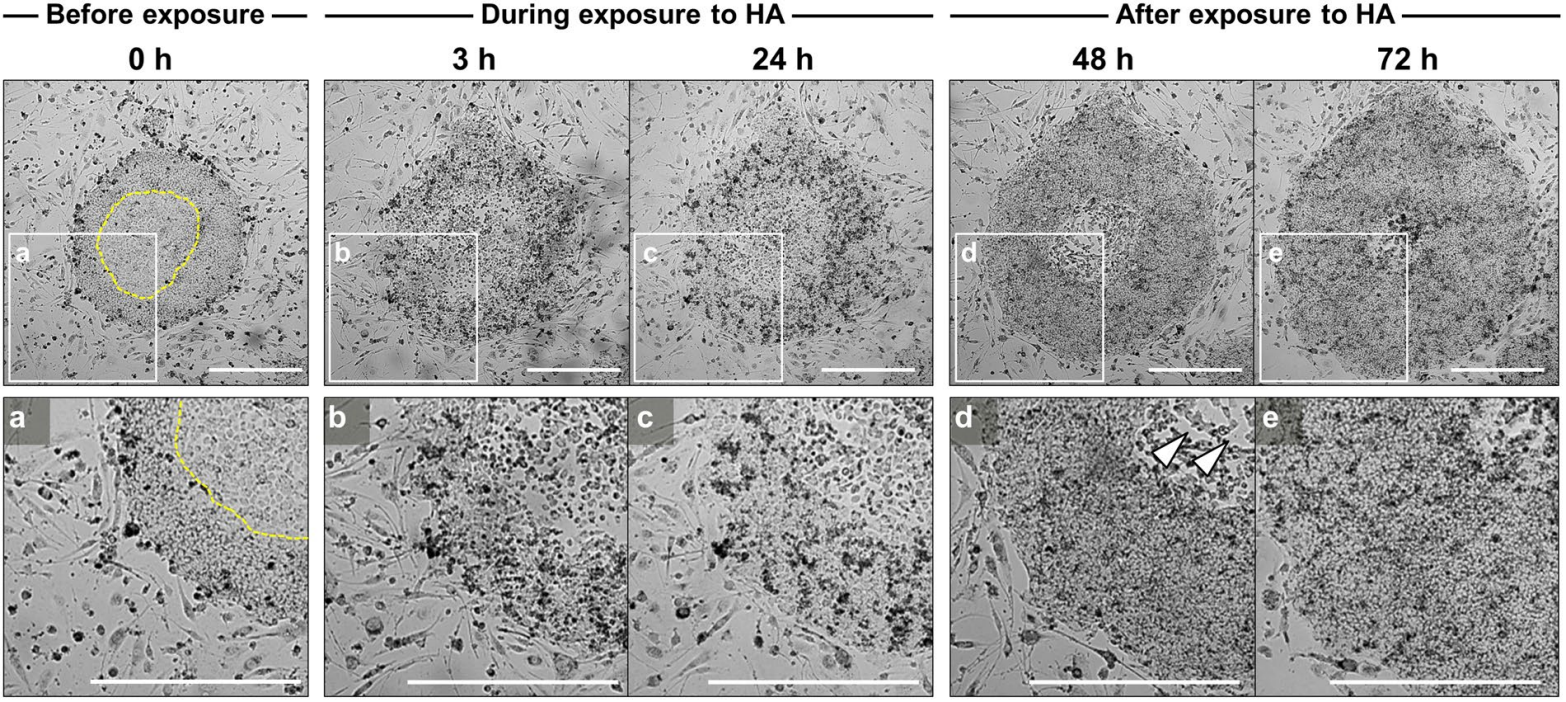
HA-mediated removal of deviated cells from hiPSC colonies exhibiting deviation in culture with SNL feeder cells. Time-lapse sequences of single hiPSC colonies after exposure to HA. After 24 h HA treatment, colonies containing deviated cells in central regions lost their adhesions with neighboring cells and shrank, with most detaching from the substrate. Dotted lines indicate boundaries between undifferentiated region and deviated region in the hiPSC colony. The areas outlined by boxes are shown at higher magnification in the lower panels (a–e). Solid arrowheads indicate the regions where deviated cells, which were exclusively observed in hiPSC colonies, had been removed. The vacated space inside colonies became filled by dividing neighboring cells, which, like undifferentiated cells, formed tight compact colonies. Scale bars = 1 mm. See Supplementary Video 1. The images are representative of >10 independent experiments that gave similar results.

To understand the HA-induced behavioral changes in hiPSC colonies, we performed time-lapse imaging on representative colonies cultured with SNL feeder cells. We cultured hiPSCs with SNL feeder cells for 72 h and then added 100 nM HA to colonies that contained deviated cells at the center and periphery; after 24 h, we replaced the medium with fresh hiPSC culture medium. At 24 h after HA treatment, colonies containing deviated cells in central regions lost their adhesions with neighboring cells and shrank, which most of these detaching from the substrate (Fig. 2 and Supplementary Video 1). Following routine medium change, the vacated space inside the colonies became filled with dividing neighboring cells, which formed tight compact colonies like those formed by undifferentiated cells. In contrast to colonies displaying deviation, colonies containing only undifferentiated cells exhibited a loose morphology initially after HA exposure, but became increasingly compact as colonies expanded during culture (Supplementary Video 2). Immunofluorescence staining showed that all cells in the colonies were OCT3/4 positive.

Having confirmed that deviated cells detached from the surface through HA-mediated E-cadherin disruption, we next asked whether it is possible to remove the deviated cells from hiPSC colonies exhibiting deviation by experimentally disrupting E-cadherin function. We used a blocking antibody to abolish the adhesive abilities of E-cadherin, whereby hiPSCs were exposed to an anti-E-cadherin antibody, DECMA-1 for 48 h. In contrast to HA treatment, treatment of hiPSCs with the anti-E-cadherin antibody did not significantly influence cell–cell adhesion and failed to selectively remove the deviated cells in hiPSC colonies (Supplementary Fig. 1). These data indicated that effect of deviated-cell removal could only be confirmed under exposure to HA.

### Spatiotemporal localization of HA in hiPSC colonies exhibiting deviation

**Figure 3:**
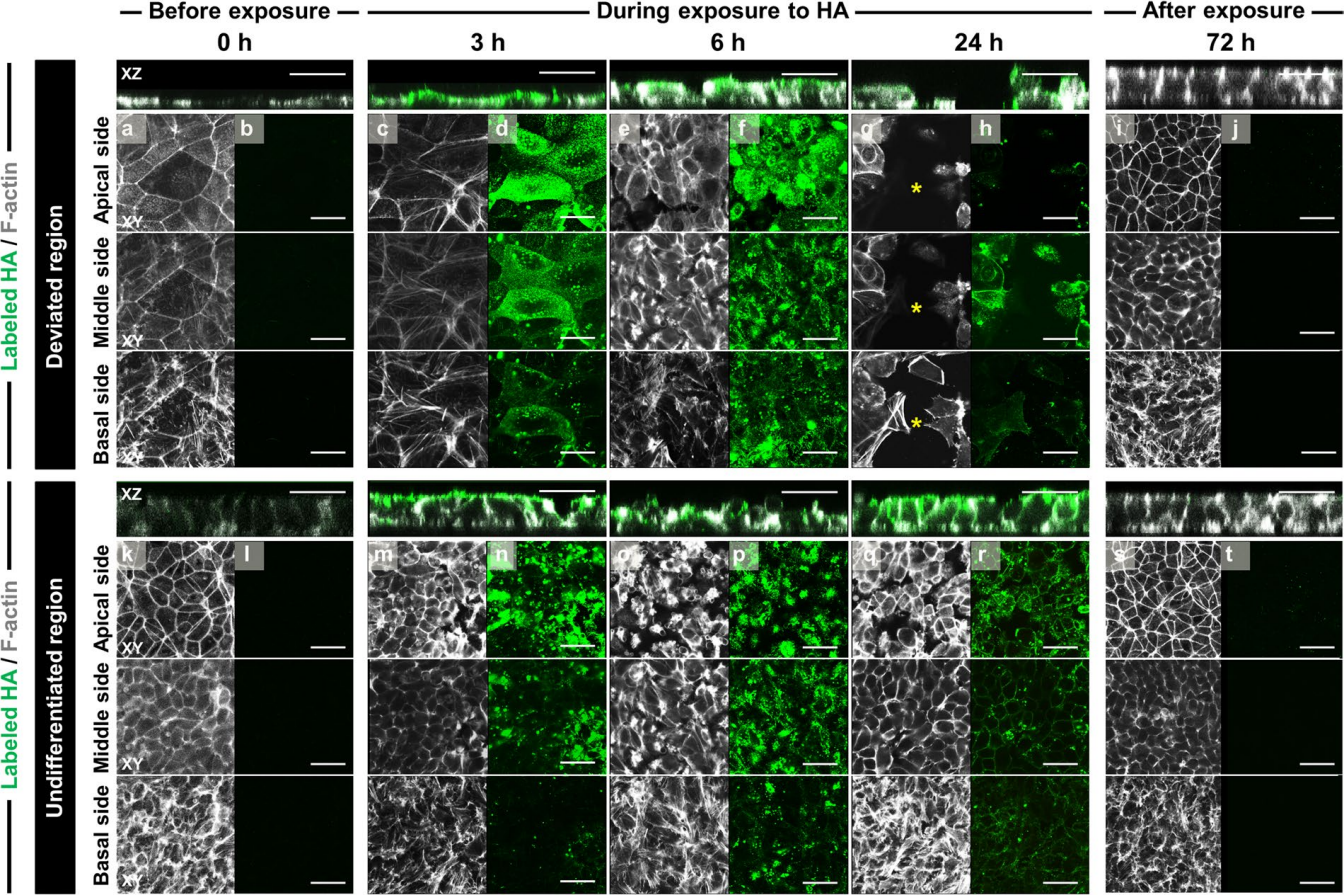
Changes in the spatiotemporal localization of labeled HA after treatment in hiPSC colonies exhibiting deviation in culture with SNL feeder cells. Confocal immunofluorescence images show 2D optical cross-sectioning (XZ and XY planes). HA (green) was localized using a Flag-M2 antibody during and after exposure to HA, and F-actin (gray) throughout the entire cell was visualized by means of phalloidin staining. Panels (a–t) show confocal images in the apical, middle and basal sides of Z-stack images. Individual confocal sections for each channel and a merged image demonstrating several examples of actin comets that co-label with HA are shown. The images show the removal process of cells deviating from the undifferentiated state in the central regions of hiPSC colonies between 6 and 24 h. Asterisks indicate the removed space of deviated cells. Cells migrated into and filled these regions as a result of the proliferation of neighboring undifferentiated cells up to 72 h. Scale bars = 50 μm.

To explore the intra- and intercellular transport of HA, we monitored the spatiotemporal behavior of HA by visualizing FLAG-tagged HA1 and HA2 with anti-FLAG-M2 antibody and staining of actin filaments. The apical side of the hiPSC colonies is defined as the top of cells facing the culture medium and basal side is defined as the side facing the substrate. Figure 3 shows a series of confocal images representing horizontal sections through the apical–basal axis of the cells in single colonies exhibiting deviation. Whereas no FLAG-M2 antibody staining was detected in hiPSCs before HA exposure (control), at 3 h after exposure, most of the fluorescent labeling was detected on the apical side of cells in both the undifferentiated region and deviated region of hiPSC colonies (Fig. 3d,n). In particular, fluorescent spots in the central regions were observed close to the basal surface (Fig. 3d). Within 24 h, almost all of the labeled HA had reached the basal side of cells and was localized at cell–cell borders at both the apical and basal surfaces. In contrast, when cultured for 72 h after HA exposure, the fluorescent spots in hiPSC colonies disappeared and labeling was negligible (similar to the control) (Fig. 3j,t).

For the spatial-temporal images of F-actin redistribution in HA-treated hiPSCs, before HA exposure, actin stress fibers were abundant along the apical and basal sides of the undifferentiated cells at the peripheral region of the colony. On the apical side, aligned actin fibers originated from the cortical actin ring and localized at cell–cell adhesions; these fibers traversed the cell lengthwise and appeared to connect intercellularly (Fig. 3k). Moreover, stress fibers spanned the entire cross-sectional area of the basal cell surface. Comparison of the central and peripheral regions inside single colonies revealed a reduction in basal-side actin fibers in the deviated cells located in the central regions (Fig. 3a). Between 3 and 6 h after HA exposure, cytoskeletal rearrangements occurred in the apical and basal sides of cells located in the center and at the periphery of colonies. At 6 h, the apical-side cortical F-actin was disrupted in undifferentiated cells, but basal-side stress fibers appeared to be unaffected (Fig. 3o). Conversely, F-actin-fiber levels in both the apical and basal sides of deviated cells located in central regions were decreased substantially: at 3 h, shortened and disorganized actin filaments were detected; at 6 h, stress fibers had disappeared (Fig. 3c,e). At 24 h, we observed that F-actin-free cell-surface membrane blebs, in which the membrane appeared to have detached from the underlying cortical-actin layer, coexisted in single cells with morphologically similar blebs with F-actin enriched at the outer membrane. After 24 h HA exposure, F-actin relative intensity in undifferentiated cells was diminished, but this gradually recovered with time. The cells largely restored their pre-HA-exposure structures in both central and peripheral regions of hiPSC colonies, and as the cells became more tightly packed, their height increased (Fig. 3i,s).

### Redistribution of E-cadherin-mediated cell–cell and focal adhesions after HA exposure in hiPSC colonies exhibiting deviation

**Figure 4:**
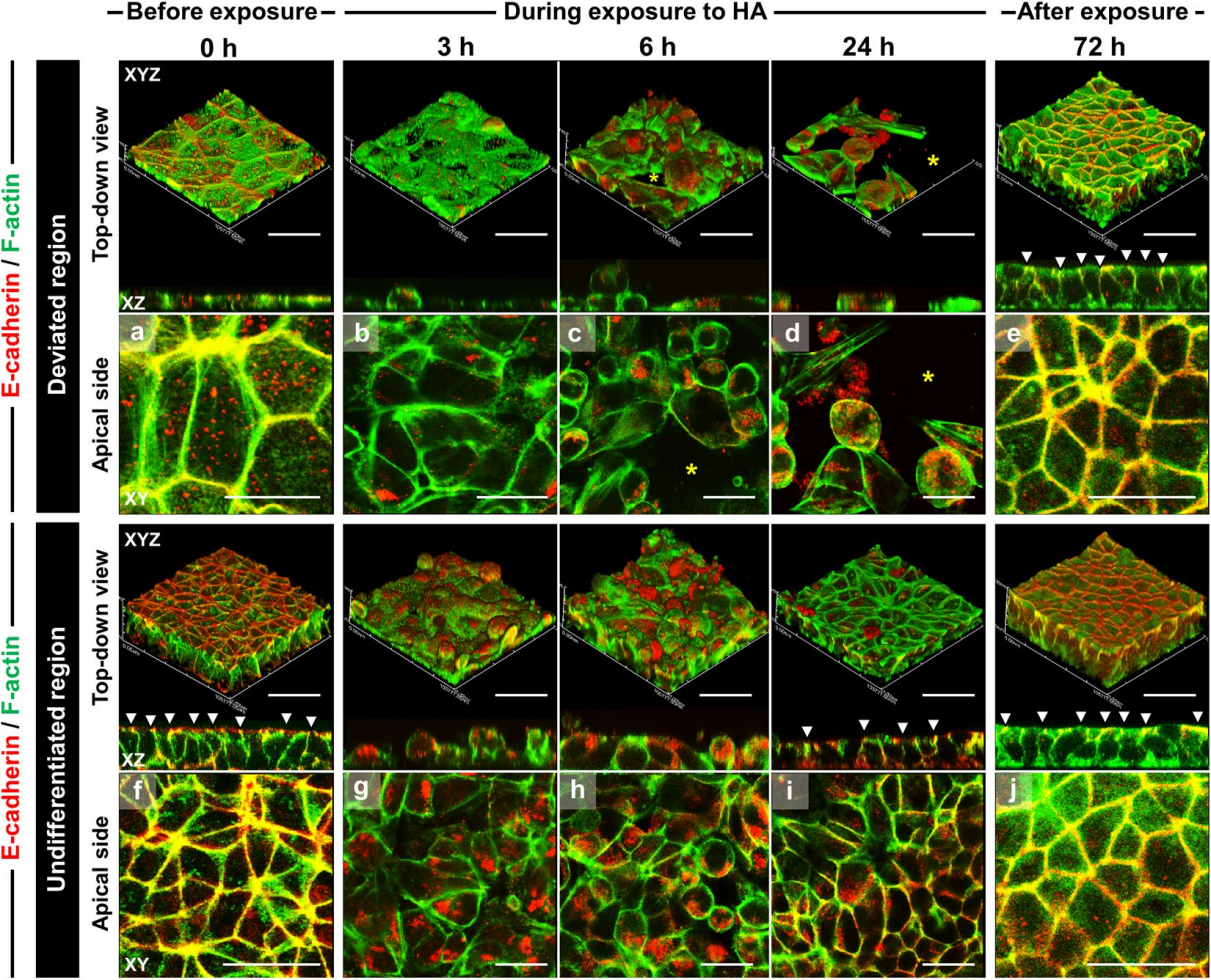
Reorganization of E-cadherin associated with the actin cytoskeleton in hiPSC colonies exhibiting deviation in culture with SNL feeder cells after HA exposure. Confocal immunofluorescence images show top-down views of 3D-reconstruction (XYZ planes) and 2D optical cross-sectioning (XZ and XY planes). Panels (a–j) are magnified in the images of apical side with a top-down view. Solid arrowheads in XZ-sections indicate the E-cadherin (red) and F-actin (green) overlap sites. The images show the removal process of cells deviating from the undifferentiated state in the central regions of hiPSC colonies between 6 and 24 h. Asterisks indicate the removed space of deviated cells. Cells migrated into and filled these regions as a result of the proliferation of neighboring undifferentiated cells up to 72 h. Scale bars = 50 μm.

Next, we tested whether HA exposure alters E-cadherin-mediated cell–cell adhesion and concomitantly disrupts actin filaments in hiPSC colonies. HA treatment triggered E-cadherin and F-actin reorganization in hiPSCs, in both time- and location-dependent manners. Before HA exposure, E-cadherin expression markedly differed in undifferentiated and deviated regions of colonies (Fig. 4). Deviated cells in central regions that aberrantly expressed E-cadherin appeared large and occupied a dispersed region, and their staining pattern appeared discontinuous and fragmented compared with cells in the periphery. Between 3 and 6 h after HA exposure, E-cadherin levels appeared downregulated after HA translocation: at 3 h, cortical actin remained in the apical part of cells, but E-cadherin was absent at certain cell–cell contacts; at 6 h, E-cadherin staining was broadly observed in dot-like structures or in extremely short fragments at cell–cell borders, and was lost in all but a few cells. By 24 h, deviated cells at the center of colonies detached from the surface, whereas the undifferentiated cells at the periphery exhibited restoration of E-cadherin-mediated cell–cell adhesion. After 24 h HA exposure, E-cadherin staining appeared as a mixture of dotted and continuous lines, which indicated that the spot-like cadherin-mediated cell–cell adhesions had begun to fuse side-by-side to form “belt-like” cell–cell adhesions. The circular actin-filament bundles thickened and expanded towards the cell periphery, and, concomitantly, stress-fiber bundles originating at regions of E-cadherin-mediated cell–cell adhesion became shortened. This staining pattern completely resembled that of non-HA-treated cells.

**Figure 5:**
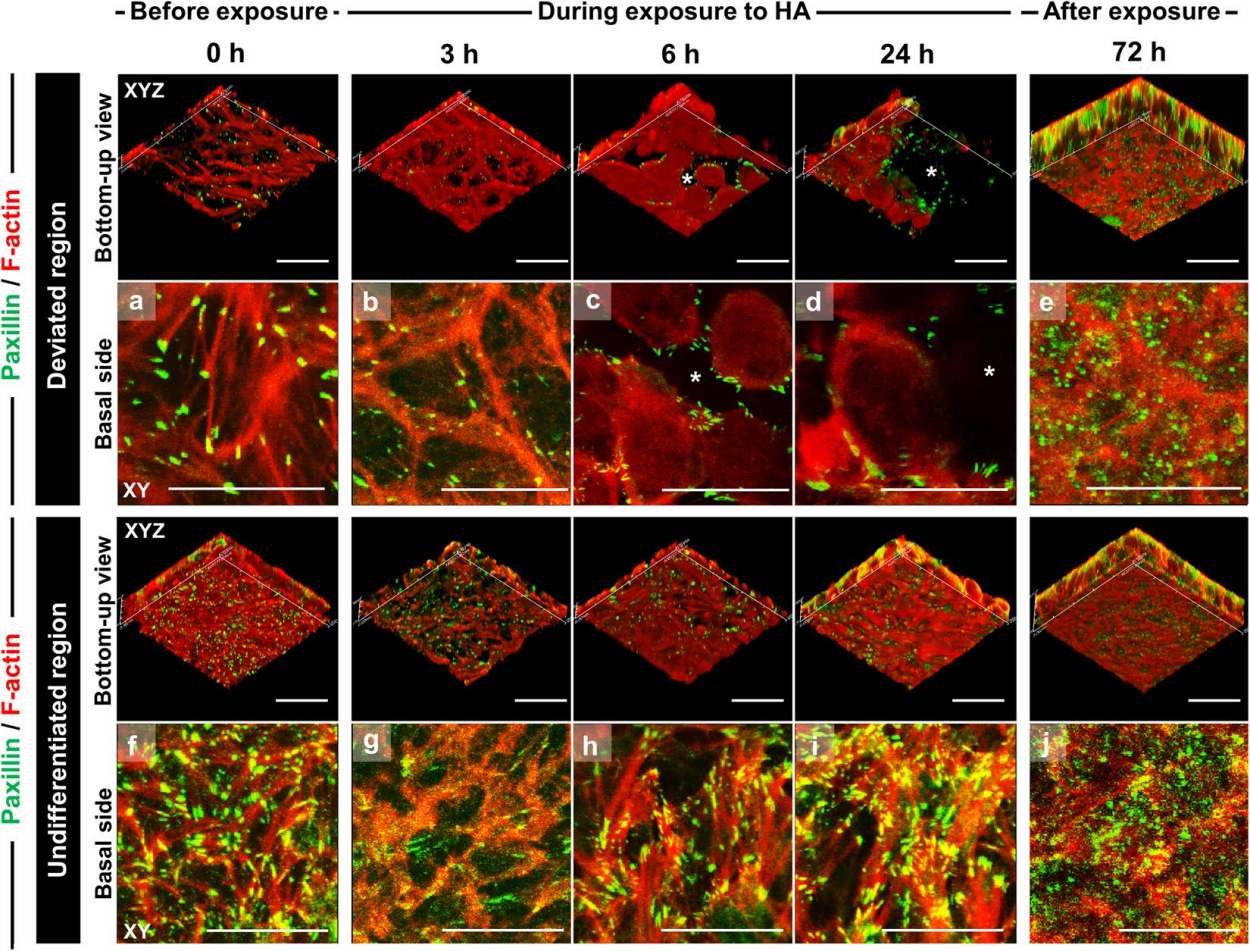
Focal-adhesion reorganization associated with cytoskeletal formation in hiPSC colonies exhibiting deviation in culture with SNL feeder cells after HA exposure. Confocal immunofluorescence images show bottom-up views (XYZ planes) of 3D-reconstruction and 2D optical cross-sectional views (XY planes). Panels (a–j) are magnified in images of basal side with a top-down view. F-actin/paxillin overlap sites appear yellow in images of basal side. The images show the removal process of cells deviating from the undifferentiated state in the central regions of hiPSC colonies between 6 and 24 h. Asterisks indicate the removed space of deviated cells. Cells migrated into and filled these regions as a result of the proliferation of neighboring undifferentiated cells up to 72 h. Scale bars = 50 μm.

We also examined how HA affected actin-cytoskeleton organization, focal-adhesion and intercellular-adhesion integrity, and finally, cell detachment. Before HA exposure, in the undifferentiated cells at peripheral regions of a colony, many of the spots of paxillin, a focal adhesion protein were distributed throughout the cytoplasm and colocalized with F-actin at stress-fiber ends (Fig. 5f). Some of the stress fibers were crisscrossed, while in others, actin fibers occasionally connected to single focal adhesions; this occurred at both cell-to-cell appositions and basal-surface focal adhesions. Both phenomena were seldom observed in deviated cells in central regions of a colony, while stress fibers and paxillin spots were primarily localized at cell edges and were fewer than in undifferentiated cells (Fig. 5a).

HA exposure induced considerable redistribution and reorganization of the actin cytoskeleton and focal adhesions in hiPSC colonies displaying deviation. At 3 h HA exposure, actin stress fibers and paxillin spots in the deviated cells in colony centers were drastically diminished in both the cell body and the cortex (Fig. 5b). At 6 h HA exposure, stress fibers were absent in the cell body, and their abundance and size were markedly decreased in the cortex (Fig. 5c). After 24 h HA exposure, actin-fiber and paxillin organization at the basal surface were disrupted, and remnants of both structures were aggregated in randomly dispersed clusters (Fig. 5d). Notably, paxillin did not colocalize with F-actin in apical-surface cytoplasmic blebs in these deviated cells, but were localized partially at cell periphery. In contrast, in undifferentiated cells at the periphery of colonies, actin filaments and paxillin at the basal surface were not clearly affected by HA exposure (Fig. 5i). After more than 24 h HA exposure, in the undifferentiated regions, both paxillin recruitment to focal adhesions and actin-bundle formation increased at the basal surface of cells. At 72 h after HA exposure, apical cortical-actin organization was completely restored and cells exhibited a clear hiPSC morphology and resembled non-HA-treated cells (Fig. 5j).

### Coordinated E-cadherin-Rap1 interplay in hiPSC colonies after HA exposure

**Figure 6:**
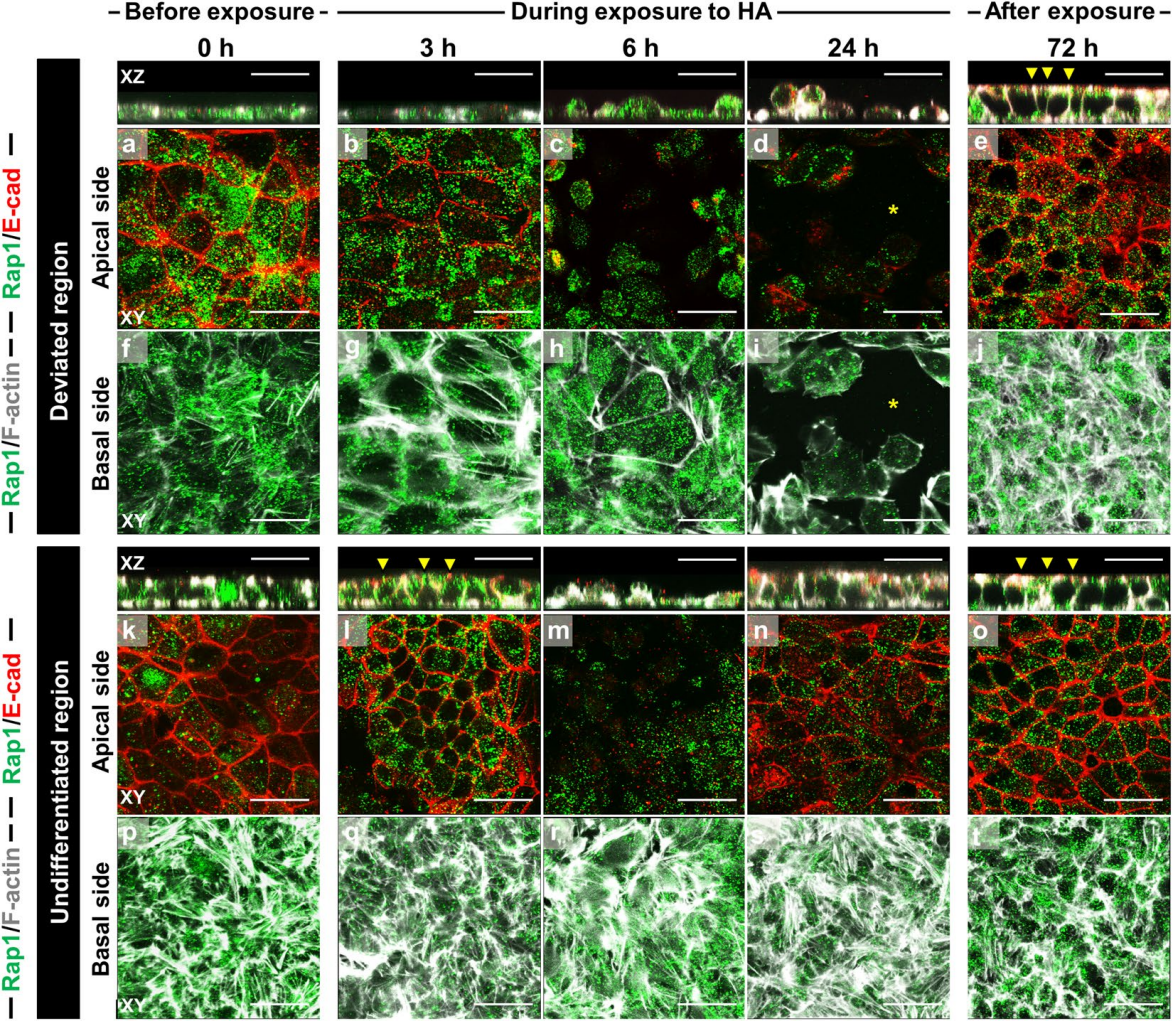
Recovery of E-cadherin-mediated cell junction formation through Rap1 signaling in hiPSC colonies exhibiting deviation in culture with SNL feeder cells after HA exposure. Confocal immunofluorescence images show the localization of Rap1 (green) and E-cadherin (red) in hiPSC colonies. Phalloidin staining shows F-actin (gray) throughout the entire cell. 2D optical cross-sectional views (XY planes) show the merged images with Rap1, E-cadherin, and F-actin. Solid arrowheads in side view point to Rap1, E-cadherin, and F-actin-positive spots. Panels (a–t) are merged images with Rap1/E-cadherin (a–e and k–o) of apical side and Rap1/F-actin (f–j and p–t) of basal side, respectively. Images show the removal process of cells deviating from the undifferentiated state in the central regions of hiPSC colonies between 6 and 24 h. Asterisks indicate the removed space of deviated cells. Cells migrated into and filled these regions as a result of the proliferation of neighboring undifferentiated cells up to 72 h. Scale bars = 50 μm.

To determine whether Rap1 functions in E-cadherin recruitment to cell–cell adhesions after HA exposure, we immunostained for E-cadherin and Rap1 and compared the spatiotemporal changes in their localizations. Comparison of undifferentiated and deviated regions in colonies after HA exposure revealed that in undifferentiated cells, Rap1 was enriched on the lateral sides where cell-cell junctions connect neighboring cells and distributed occasionally basal sides of cells (Fig. 6). After 24 h HA exposure, Rap1 was localized at perinuclear regions and on the plasma membrane in deviated cells, which had occasionally detached from the surface (Fig. 6i). In undifferentiated regions at the colony periphery, Rap1 colocalized with E-cadherin at nascent cell–cell adhesion sites, such as at “zipper-like” structures at 24 h HA exposure; this suggests that Rap1 is recruited to somewhat mature cell–cell adhesions (Fig. 6n). Rap1 and E-cadherin colocalized at adhesion sites at 72 h after HA exposure, with Rap1 also being enriched along the lateral sides of cells (Fig. 6e,o). To examine whether Rap regulate the hiPSC morphological recovery and deviated-cell detachment following HA exposure, we treated hiPSCs with the Rap1 inhibitor GGTI-298 (15 and 25 μM) or Rap1 activator 8-CPT-2Me-cAMP (1 and 4 μM). Combined treatment of HA with the Rap1 inhibitor GGTI-298 (15 μM) delayed hiPSC morphological recovery and accelerated deviated-cell detachment (Supplementary Fig. 2). Notably, HA-treated cells exposed to high Rap1-inhibitor concentration (25 μM) noticeably detached from the surface within 24 h. However, combined treatment of HA with the Rap1 activator 8-CPT-2Me-cAMP did not result in any increase in deviated-cell detachment. Rap1 activator also did not lead to further deviated-cell detachment, although the deviated region show a loss of cell–cell adhesion following HA exposure. These data indicate that Rap1 is required for recruiting E-cadherin to nascent cell–cell adhesions and actin reorganization after HA exposure, and not for maintaining E-cadherin at mature cell–cell adhesions.

## Discussion

**Figure 7:**
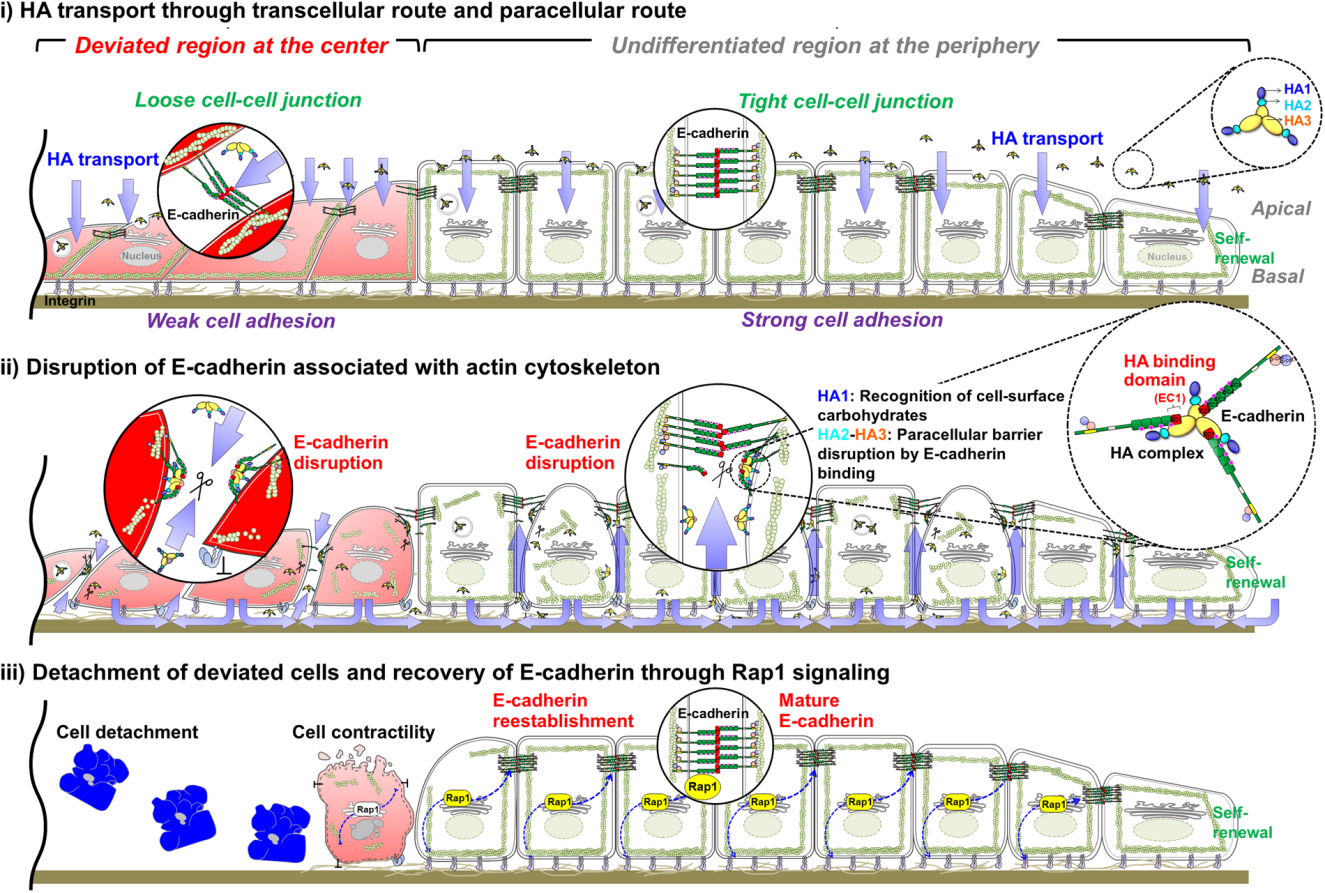
Schematic showing our hypothesis on the mechanism by which HA removes deviated cells from undifferentiated hiPSC colonies exhibiting deviation in culture with SNL feeder cells. The first extracellular cadherin domain (EC1), which mediates cell–cell interactions in trans, appears to be critical for HA-cadherin association. Based on the result of this study, we constructed the diagram in line with the following assumptions: (i) HA can pass through cells (transcellular route) and between cells (paracellular route) when moving across cells. (ii) HA binds to E-cadherin and thereby disrupts E-cadherin-mediated cell–cell adhesion. (iii) Loss of E-cadherin alters cytoskeletal organization and focal adhesion by regulating the activity of Rap1, which functions in the interplay between cadherin and integrin. Undifferentiated cells can completely recover the structures and functions of E-cadherin and integrin in hiPSC colonies because of their ability to activate Rap1.

In this study, we focused on the mechanisms underlying HA-mediated selective elimination of cells that deviate from the undifferentiated state in hiPSC colonies. Figure 7 illustrates our working hypothesis based on the results obtained in this study with two main questions being addressed. First, how does HA access E-cadherin and disrupt E-cadherin-mediated cell–cell adhesion in hiPSC colonies? HA can be transported via the transcellular route (across cells) and/or paracellular route (between cells)^31^. HA complexes bind to E-cadherin with high specificity, which involves extensive intermolecular interactions, and to cell-surface carbohydrates^31, 32^, and they disrupt E-cadherin-mediated cell–cell adhesion. HA-induced E-cadherin disruption alters the structural and functional tightness of the barrier, depending on cell type. Herein, we observed that HA distribution markedly differed between deviated and undifferentiated cells and correlated with differences detected in their E-cadherin expression. The intact structure of undifferentiated cells was maintained by the integrity of the apical-basal polarity and highly organized E-cadherin linked to the actin cytoskeleton (Fig. 4). In comparison, in the central region within single colonies exhibiting deviation, the deviated cells were large and dispersed, and their staining pattern appeared discontinuous and fragmented. Furthermore, E-cadherin was aberrantly clustered at residual cell–cell adhesions, with a considerable gap separating E-cadherin-enriched cell–cell-adhesion regions from circumferential actin-filament bundles. Analysis of HA translocation revealed that more HA passed freely, both within a cell and across adjacent cells (paracellular space) in central regions of colonies than between undifferentiated cells (Fig. 3). This subsequently weakened E-cadherin-mediated cell–cell adhesions and resulted in rapid HA translocation via the paracellular route, suggesting that the disruption of E-cadherin-mediated adhesion was facilitated. Likewise, addition of the DECMA-1 antibody, directed against the extracellular domain of E-cadherin, did not significantly influence cell–cell adhesion. Taken together, these data indicate that endocytic activities of HA facilitate selective removal of deviated cells.

Second, how does HA exposure selectively remove cells that deviate from the undifferentiated state in hiPSC colonies? This removal might depend on the difference in strength of E-cadherin-mediated adhesion linked to the actin cytoskeleton, and the distinct abilities of cells to recover after HA-induced E-cadherin disruption between the two regions in hiPSC colonies exhibiting deviation. HA treatment disrupted E-cadherin binding at cell–cell adhesions and triggered drastic reorganization and disruption of the actin cytoskeleton and focal adhesions in both regions of hiPSC colonies exhibiting deviation. Within 6 h of HA exposure, the E-cadherin ring-like structure disappeared; the organization of the adhesion belts delineated by E-cadherin lost their circular F-actin arrangement, and basal-surface stress fibers and focal adhesions decreased (Figs 4 and 5). The deviated cells in central regions appeared highly rounded up, indicating that cytoskeleton-mediated cell contraction occurred before cell detachment. Because hiPSCs individually generate actomyosin contractile stress, each intercellular adhesive junction is subjected to the opposed stresses independently generated by its two partner cells^26, 27^. It is assumed that, like hESCs, the actin cytoskeleton in hiPSCs connects cell–cell junctions to focal adhesions and consequently, changes in cytoskeletal tension directly affect barrier structure and function^26^. After loss of cell–cell adhesion, myosin-actin-dependent contraction leads to cell death; conversely, cell–cell adhesion promotes survival by inhibiting this contraction^27–30^. Thus, integrin-dependent actin-cytoskeleton contraction and the physical breakdown of integrin might facilitate the detachment of deviated cells from the surface after HA exposure. In contrast, in undifferentiated hiPSCs, basal-surface stress fibers and focal adhesions were maintained, although E-cadherin linkage to the actin cytoskeleton was disrupted after HA exposure (Fig. 5). E-cadherin-associated cytoskeletal structures were fully restored at both apical and basal surfaces at 72 h after HA exposure. The actin fibers became radially organized and terminated at E-cadherin puncta, and morphologically, this was reminiscent of the zipper-like junctions observed at nascent cell–cell adhesions. Previously, Rap1 activation was proposed to regulate barrier function by increasing E-cadherin expression and inducing changes in the actin cytoskeleton and focal adhesions^16–20, 29^. Rap1 activation triggered by E-cadherin endocytosis after E-cadherin disruption is required to enhance integrin-dependent cell–substrate adhesion^29^. We similarly found that Rap1 coordinates E-cadherin-integrin interplay in hiPSCs: Rap1-dependent coordinated modulation of E-cadherin and integrin functions associated with cytoskeleton reorganization plays an essential role in restoring hiPSC structure and function after loss of E-cadherin binding. Concurrent HA and Rap1-inhibitor treatment delayed the recovery of undifferentiated hiPSC morphology; however, deviated cells detached faster compared with untreated controls (Supplementary Fig. 2). However, combined treatment of HA with Rap1 activator failed to remove the deviated cells, and this failure correlated with these activators’ inability to induce E-cadherin recycling after loss of cell–cell adhesion. This finding indicates that in the two regions in hiPSC colonies, the difference in response exhibited in HA-induced disruption of E-cadherin-mediated cell–cell adhesion depends on the ability of cells to recover E-cadherin structure and function through the actions of actin-cytoskeleton components.

## Conclusion

The present study suggests that the different adhesion states that produce variations in adhesion strength and E-cadherin turnover between undifferentiated and deviated regions in hiPSC colonies correlated with the selective removal of deviated cells through E-cadherin disruption. In deviated cells exhibiting weak E-cadherin adhesion in the center of hiPSC colonies, HA-induced E-cadherin disruption can facilitate HA transport to E-cadherin, thereby promoting deviated-cell removal. However, the undifferentiated cells ability to regenerate E-cadherin can maintain hiPSCs in an undifferentiated state after disruption of E-cadherin binding. Rap1-mediated positive-feedback regulation could lead to E-cadherin re-establishment at intercellular junctions and thereby enable undifferentiated cells to recover intercellular association and colony structure. Our results suggest a robust and stable hiPSC culture-propagation strategy that involves HA-mediated selective removal of cells that deviate from the undifferentiated state. This technology opens new avenues for selectively removing deviated cells that aberrantly express E-cadherin in hiPSC colonies during culture, and it facilitates rational design of culture strategies for maintaining undifferentiated hiPSCs.

## Methods

### Cells and culture conditions

The hiPSC line Tic (provided by JCRB1331, JCRB Cell Bank, Osaka, Japan) was routinely maintained on mitomycin C-treated SNL76/7 cells (European Collection of Cell Cultures, Salisbury, UK) in commercially available medium (ReproStem, ReproCELL Inc., Tokyo, Japan) supplemented with 5 ng/mL basic fibroblast growth factor. For routine passaging of hiPSCs, feeder cells were removed after incubation for 1 min with CTK solution as previously described^8^. When SNL cells detached and the edges of hiPSC colonies started curling, the dissociation solution (CTK; 0.1 mg/mL collagenase IV, 0.25% trypsin, 0.1 mM CaCl_2_) was removed and cells were washed once with culture medium. hiPSC colonies in the undifferentiated state were carefully collected using a cell scraper (Sumitomo Bakelite Co. Ltd., Osaka, Japan). Suspensions of collected undifferentiated colonies were gently pipetted to disperse them into small aggregates, and were subsequently dispensed into a fresh culture vessel containing feeder cells. Cells were then collected by gentle pipetting, and transferred to a new dish pre-coated with SNL76/7 cells.

For preparing feeder layers, mitomycin C-treated SNL76/7 cells were cultured in advance for 24 h in Dulbecco’s modified Eagle’s medium (DMEM; Sigma-Aldrich, St. Louis, MO, USA) supplemented with 7% fetal bovine serum (FBS; Life Technologies, Grand Island, NY, USA) and antibiotics (100 U/cm^3^ penicillin G, 0.1 mg/cm^3^ streptomycin, and 0.25 mg/cm^3^ amphotericin B; all obtained from Life Technologies). The seeding density was fixed at a viable cell concentration of 2.5 × 10^4^ cells/cm^2^.

### Reconstitution of the functional HA complex

The procedure used for reconstituting the functional HA complex was similar to that described previously^32^. Briefly, we expressed each subcomponent of HA separately in *Escherichia coli* and purified them; when the three subcomponents were mixed and incubated at 37 °C, a large molecular complex formed spontaneously. We achieved functional reconstitution of entire HA (HA1–HA2–HA3) by using purified recombinant HA1, HA2, and HA3 of serotype B. The purified proteins were dialyzed against PBS, pH 7.4. Protein concentrations were determined using the BCA Protein Assay Reagent (Thermo Scientific, Rockford, IL, USA).

### HA-exposure conditions

We cultured hiPSCs on SNL feeder cells for 3 days and then exposed them to 100 μM HA. HA was removed in the course of routine medium changes, and the behavioral changes in undifferentiated and deviated cells in hiPSC colonies were examined before, during, and after HA exposure for 24 h. All measurements were obtained in both control and HA-treated cultures.

### Inhibition of cell–cell adhesion using a blocking antibody

We seeded hiPSCs on SNL feeder cells and cultured them in hiPSC medium, as already described. At 3 days, the anti-E-cadherin antibody DECMA-1 (Zymed Laboratories, San Francisco, CA, USA) was added at a final concentration of 20 μg/mL. The antibody was reapplied every second day with each media change.

### Regulation of cell–cell adhesion through Rap1 inhibition or activation

To examine the effect of Rap1 on the recovery of hiPSC morphology after HA exposure, cells were co-treated with (as indicated) 100 μM HA, and 15 or 25 μM Rap1 inhibitor (geranylgeranyltransferase I inhibitor, GGTI-298; Calbiochem, Merck Chemicals Ltd., Nottingham, UK) as well as 1 or 4 μM Rap1 activator (an Epac agonist, 8-CPT-2Me-cAMP; Merck Millipore, Darmstadt, Germany) and cultured for 72 h.

### Time-lapse live-cell imaging

To investigate the dynamics of the changes in cell morphology, during and after exposure to HA, cells were positioned on an image analyzer (In CELL Analyzer 2000; GE Healthcare, Buckinghamshire, UK), and observed through a 4× objective lens, with images obtained every 1 h at several positions.

### Immunofluorescence staining

The procedure used for immunofluorescent staining was similar to that described previously^36^. Briefly, hiPSCs were fixed with 3.7% paraformaldehyde (Wako Pure Chemical Industries, Osaka, Japan) for 10 min at room temperature and then rinsed with PBS, after which they were soaked for 4 min in PBS containing 0.25% Triton X-100. After masking nonspecific proteins by incubation in Block Ace (Dainippon Sumitomo Pharma Co., Ltd., Osaka, Japan) for 1 h at ambient temperature, the cells were incubated with the following primary antibodies (diluted adequately in PBS containing 10% Block Ace): anti-OCT3/4 (Santa Cruz Biotechnology, Santa Cruz, CA, USA); anti-NANOG, anti-SSEA-4, and anti-TRA-l-60 (Stemgent, San Diego, CA, USA); anti-SSEA-1 (BD Biosciences Pharmingen, San Diego, CA, USA); anti-βIII-tubulin, anti-α-fetoprotein, and anti-FLAG M2 (Sigma-Aldrich); anti-desmin, anti-α-smooth muscle actin, and anti-Rap1 (Abcam, Cambridge, MA, USA); anti-paxillin (clone 15D2, Zymed/Invitrogen); and anti-E-cadherin (Takara Biomedicals, Shiga, Japan) at 4 °C overnight. Cells were washed with Tris-buffered saline and then immunolabeled with Alexa Fluor 488-conjugated goat anti-rabbit or Alexa Fluor 594-conjugated goat anti-mouse IgG (Life Technologies) for 1 h. F-actin and cell nuclei were stained with Alexa Fluor 633-conjugated phalloidin (Life Technologies) and 4′,6-diamidino-2-phenylindole (DAPI; Life Technologies), respectively. Images were obtained using a confocal laser scanning microscope (Model FV-1000; Olympus, Tokyo, Japan), with a 60× objective lens and an image analyzer and a 10× objective lens, under fluorescence excitation at 358, 488, 594, and 633 nm.

## Electronic supplementary material

Supplementary Material
