# Supplementary material for: Botulinum hemagglutinin-mediated selective removal of cells deviating from the undifferentiated state in hiPSC colonies
Source: Sci Rep. 2017 Mar 7;7:93. doi: 10.1038/s41598-017-00083-1 (PMC5428320; doi:10.1038/s41598-017-00083-1)
Supplement: Supplementary file 1 — Supplementary Material [file 41598_2017_83_MOESM1_ESM.pdf]

Supplementary Material

Botulinum hemagglutinin-mediated selective removal of cells deviating from the undifferentiated state in hiPSC colonies

Mee-Hae Kim [1], Yo Sugawara [2], Yukako Fujinaga [2, 3], Masahiro Kino-oka [1]*

[1]*Department of Biotechnology, Graduate School of Engineering, Osaka University, 2-1 Yamadaoka, Suita, Osaka 565-0871, Japan*

[2]*Laboratory for Infection Cell Biology, International Research Center for Infectious Diseases, Research Institute for Microbial Diseases, Osaka University, 2-1 Yamadaoka, Suita, Osaka 565-0871, Japan*

[3]*Department of Bacteriology, Graduate School of Medical Sciences, Kanazawa University, 13-1 Takara, Kanazawa, Ishikawa, 920-8641, Japan*

*To whom correspondence should be addressed. E-mail: kino-oka@bio.eng.osaka-u.ac.jp

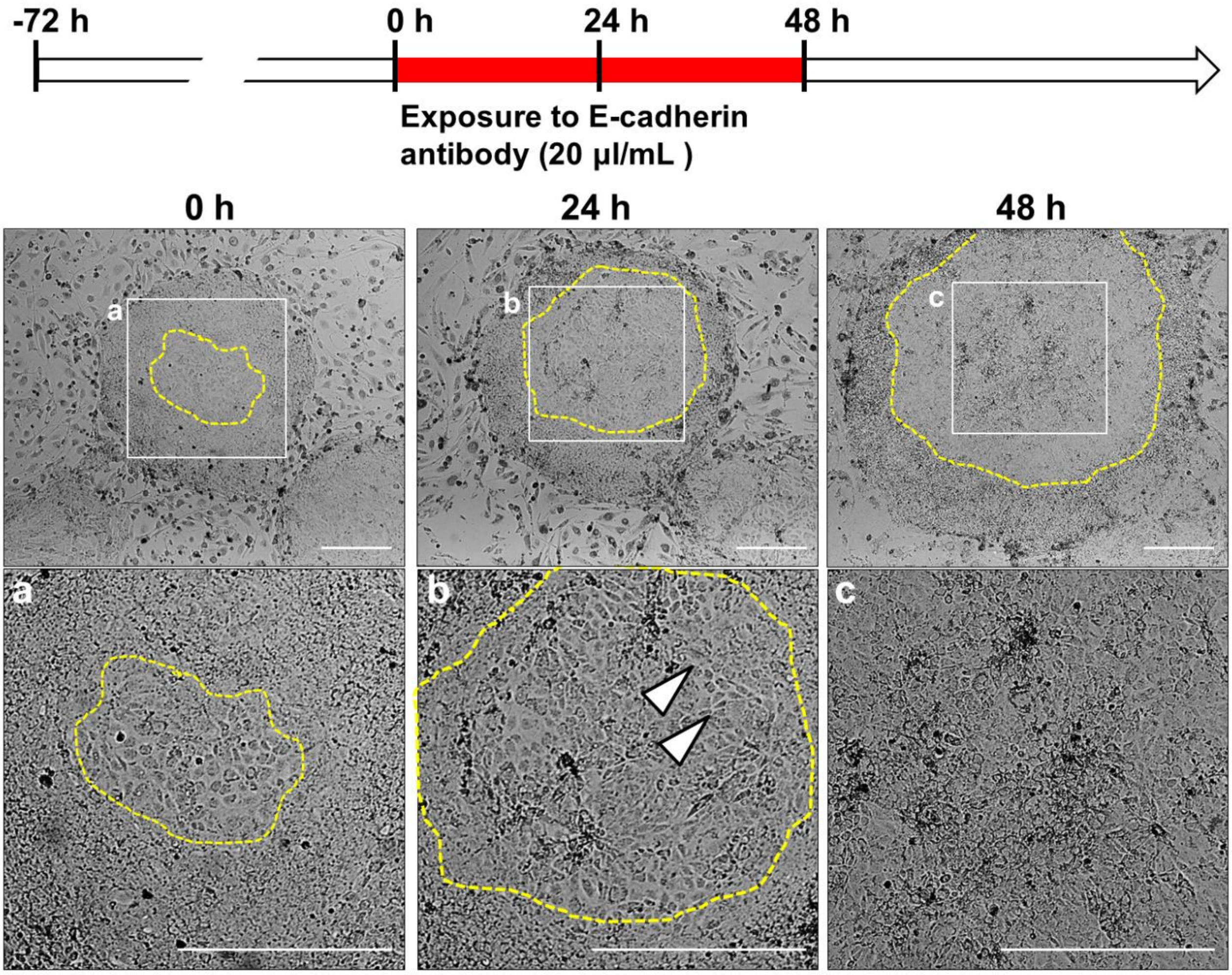

Supplementary Figure 1. Effect of cell-cell adhesion inhibitor, E-cadherin blocking antibody, on the detachment of deviated cells in hiPSC colonies. hiPSCs were cultured on SNL feeder cells for 3 days, then exposed to an E-cadherin blocking antibody for 48 h. Solid arrowheads indicate a loss of cell–cell adhesion. Dotted lines in planes indicate the boundary between undifferentiated and deviated regions in a hiPSC colony. Scale bars = 500 µm.

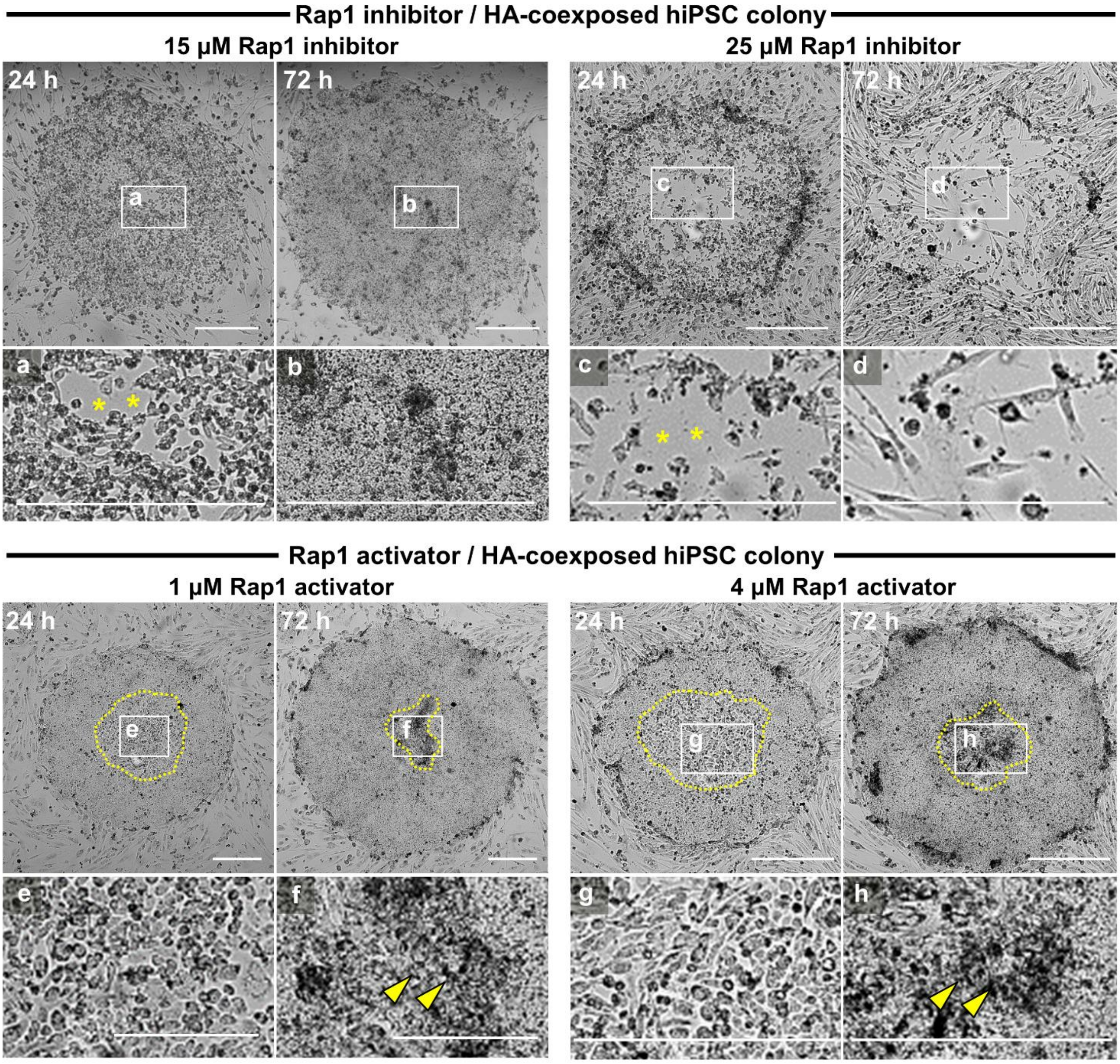

Supplementary Figure 2. Effect of Rap1 on HA-mediated selective removal of deviated cells in hiPSC colonies in culture with SNL feeder cells after exposure to HA. hiPSCs were cultured on SNL feeder cells and then co-exposed to the Rap1 inhibitor GGTI-298 or Rap1 activator 8-CPT-2Me-cAMP and HA for 24 h. Asterisks indicate the removed space of deviated cells. Solid arrowheads indicate the remaining deviated cells by a failure to detach from surface. Dotted lines in the panels indicate the boundary between undifferentiated and deviated regions in hiPSC colonies. Scale bars = 500 μm.

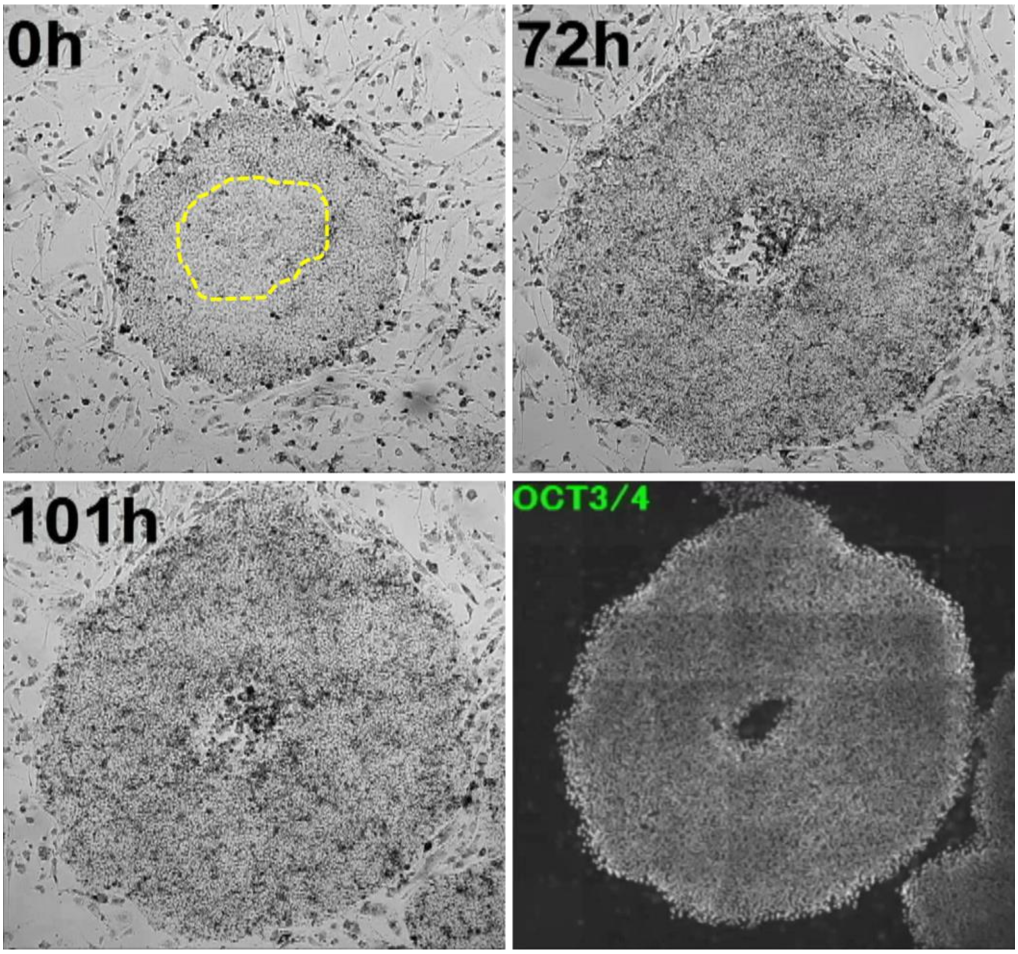

Supplementary Video 1. Time-lapse movie showing the post-HA-exposure behaviors of a hiPSC colony exhibiting deviation in culture with SNL feeder cells. Dotted lines in the panels indicate the boundary between undifferentiated and deviated regions in hiPSC colonies. In the region deviated from the undifferentiated state, HA exposure caused cells to round up, lose adhesions with their neighbors, and then detach from the culture surface. As the culture proceeded, the vacated space inside colonies was filled by dividing cells. As the culture period was lengthened, the adhesions between adjacent cells became indistinct from each other because each cell appositioned tightly against its neighbor. In contrast to the deviated cells, undifferentiated cells in the peripheral region of the colony initially rounded up, but recovered upon exposure for 72 h. Immunofluorescence staining revealed that all cells of the hiPSC colonies were positive for the pluripotency marker OCT3/4 at 101 h after exposure to HA. Figure 2 shows a representative sequence of images recorded following 24-h exposure to HA.

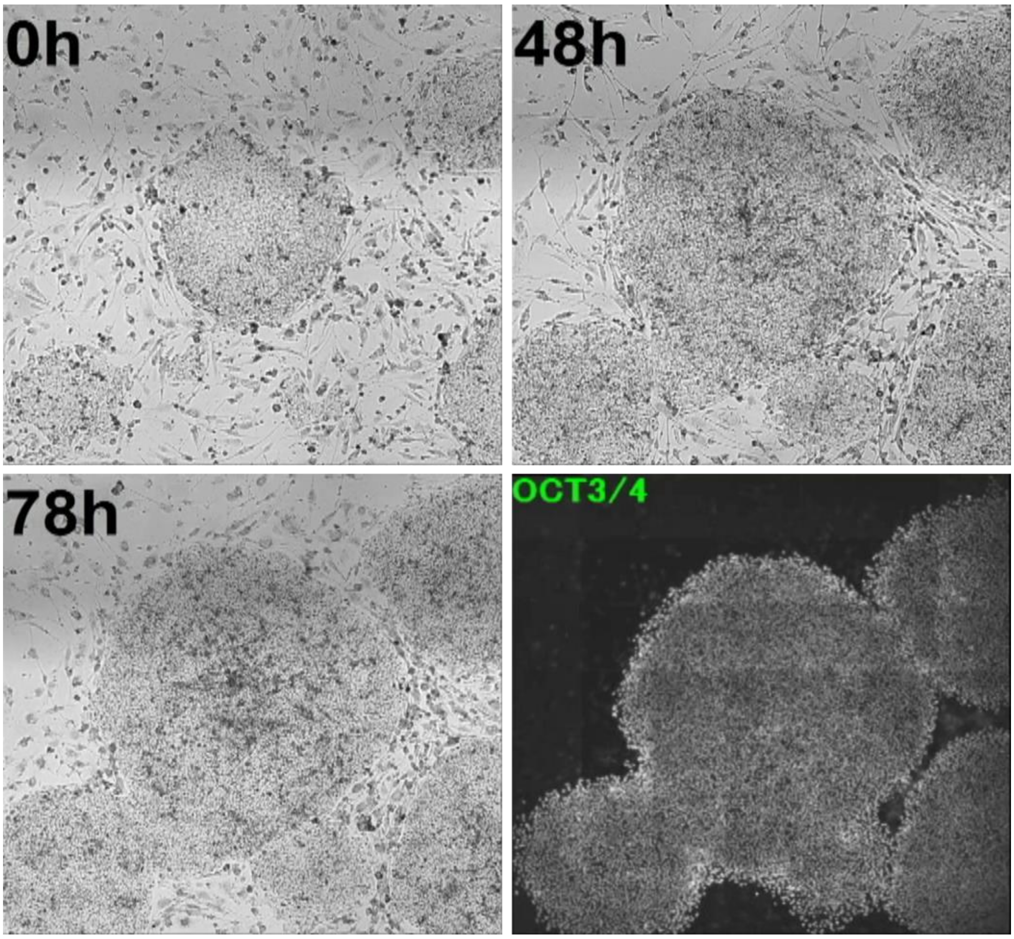

Supplementary Video 2. Time-lapse movie showing the post-HA-exposure behaviors of a hiPSC colony containing undifferentiated cells in a culture with SNL feeder cells. The undifferentiated cells initially rounded up but retained adhesions with their neighboring cells, and then recovered upon exposure for 48 h. Immunofluorescence staining revealed that all cells of the hiPSC colonies were positive for the pluripotency marker OCT3/4 at 78 h after exposure to HA.
